# Spoligotyping and drug resistance patterns of *Mycobacterium tuberculosis* isolates from five provinces of Iran

**DOI:** 10.1002/mbo3.139

**Published:** 2013-10-28

**Authors:** Mehri Haeili, Davood Darban-Sarokhalil, Abbas Ali Imani Fooladi, Sedigheh Javadpour, Abdorrazagh Hashemi, Farideh Siavoshi, Mohammad Mehdi Feizabadi

**Affiliations:** 1 Department of Microbiology, School of Biology, College of Science, University of TehranTehran, Iran; 2 Department of Microbiology, School of Medicine, Tehran University of Medical SciencesTehran, Iran; 3 Department of Pathobiology, School of Medicine, Alborz University of Medical SciencesKaraj, Iran; 4 Bagiatallah HospitalTehran, Iran; 5 Departments of Microbiology, Hormozgan University of Medical SciencesBandar Abbas, Iran; 6 Ahvaz Jundishapur University of Medical SciencesAhvaz, Iran

**Keywords:** Iran, MDR-TB, *Mycobacterium tuberculosis*, spoligotyping.

## Abstract

Tuberculosis (TB) persists as a public health problem in Iran. Characterization of *Mycobacterium tuberculosis* isolates circulating in this area will contribute to understand and control the spread of the strains. The aims of this study were to understand the genetic diversity and drug susceptibility of *M. tuberculosis* isolates circulating in Iran and to analyze the relationship between genotype and drug resistance. A total of 291 *M. tuberculosis* isolates collected from TB patients were genotyped by spoligotyping. Drug susceptibility testing was performed using proportion method. Spoligotyping resulted in 75 distinct patterns. 86.2% of isolates were grouped in 35 clusters while the remaining isolates were unique. Ural was found to be the most predominant lineage (34.3%) followed by Central Asian strain (CAS) (24%), T (18.2%), Manu2 (7.5%) and Latin American-Mediterranean (LAM) (6.1%). The five largest clusters were Ural/Spoligotype International Type (SIT)127 (15.8%), CAS1/SIT26 (9.2%), T1/SIT53 (6.1%), T1/SIT284 (5.4%), and CAS1/SIT25 (4.4%). About 5% of isolates had multidrug resistance (MDR) and 10% had other resistance. MDR was significantly associated with Beijing strains, but not with Ural family. This study highlights dominance of Ural, CAS, and T families in Iran. Biogeographic specificity of CAS and T families to border provinces of Iran including Sistan-Baluchestan and Kermanshah, respectively, suggested that this family strains might be transmitted from these regions to other provinces of the country.

## Introduction

Tuberculosis (TB) continues to be an important public health problem mainly in developing countries. It is estimated that one-third of the world's population has been infected by *Mycobacterium tuberculosis*. Each year, there are more than nine million new TB cases and almost two million deaths worldwide despite the fact that it is curable with early detection and prompt treatment (WHO 2007). Iran has a population of 75 million inhabitants and shares geographic borders with three countries where TB is endemic, that is Pakistan, Iraq, and Afghanistan. Although the reported TB incidence rate for Iran has decreased from 36/100,000 in 1990 to 17/100,000 in 2010 (WHO 2011), TB control remains a priority among public health policies. Molecular typing of *M. tuberculosis* strains is important to identify dominant strains associated with outbreak and drug resistance and also to trace the transmission chains (Bazira et al. 2011). In general, *M. tuberculosis* isolates with unique fingerprints are most commonly associated with endogenous reactivation whereas, clusters of isolates with identical deoxyribonucleic acid (DNA) patterns have been attributed to recent infection (Kaeto-Maeda and Small 2000; Viana-Niero et al. 2001). Restriction fragment length polymorphism (RFLP) analysis with *IS*6110 probe is considered as gold standard method for genotyping of *M. tuberculosis* strains (Jiao et al. 2008). However, this method is time-consuming and labor intensive. It is also unreliable for typing of strains with the low copy numbers (fewer than six) of *IS*6110 (Christianson et al. 2010). Spoligotyping is based on DNA polymorphisms within the direct repeat (DR) locus of *M. tuberculosis* complex. The DR locus consists of a series of directly repeated sequences of 36 bp, interspersed with 35–41 bp nonrepetitive spacer sequences. This method involves a polymerase chain reaction (PCR) amplification of the DR locus and hybridization to a membrane containing a series of DNA probes representing each of the unique spacer sequences in the DR locus. It is a simple and rapid method requiring much less DNA quantities than *IS*6110-RFLP (Kamerbeek et al. 1997). Furthermore, recent transfer of spoligotyping technique from membrane to a microbead format has even brought about more reliable results because of the better detection of some spacers and lower error rate in transcription and interpretation of the results. Microbead-based spoligotyping has enabled analyzing larger number of isolates per run which makes it suitable for high-throughput genotyping (Cowan et al. 2004; Zhang et al. 2010; Abadia et al. 2011).

Emergence and spread of multidrug resistant (MDR) strains of *M. tuberculosis* represent the greatest threat to TB control programs. The design of approaches for the management of MDR-TB depends on awareness of development and subsequent spread of drug-resistant isolates (Mistry et al. 2002). It is indicated that MDR-TB outbreaks have been associated with Beijing and Haarlem genotypes (Glynn et al. 2002; Mardassi et al. 2005).

The specific objectives of this study were to investigate population structure of *M. tuberculosis* isolates circulating in five provinces of Iran over a period of 2 years (2010–2012) by spoligotyping, and to evaluate the rate of drug resistance. In addition, possible association between strain type and drug resistance was also studied.

## Material and Methods

### Patient population and bacterial isolates

**Figure 1:**
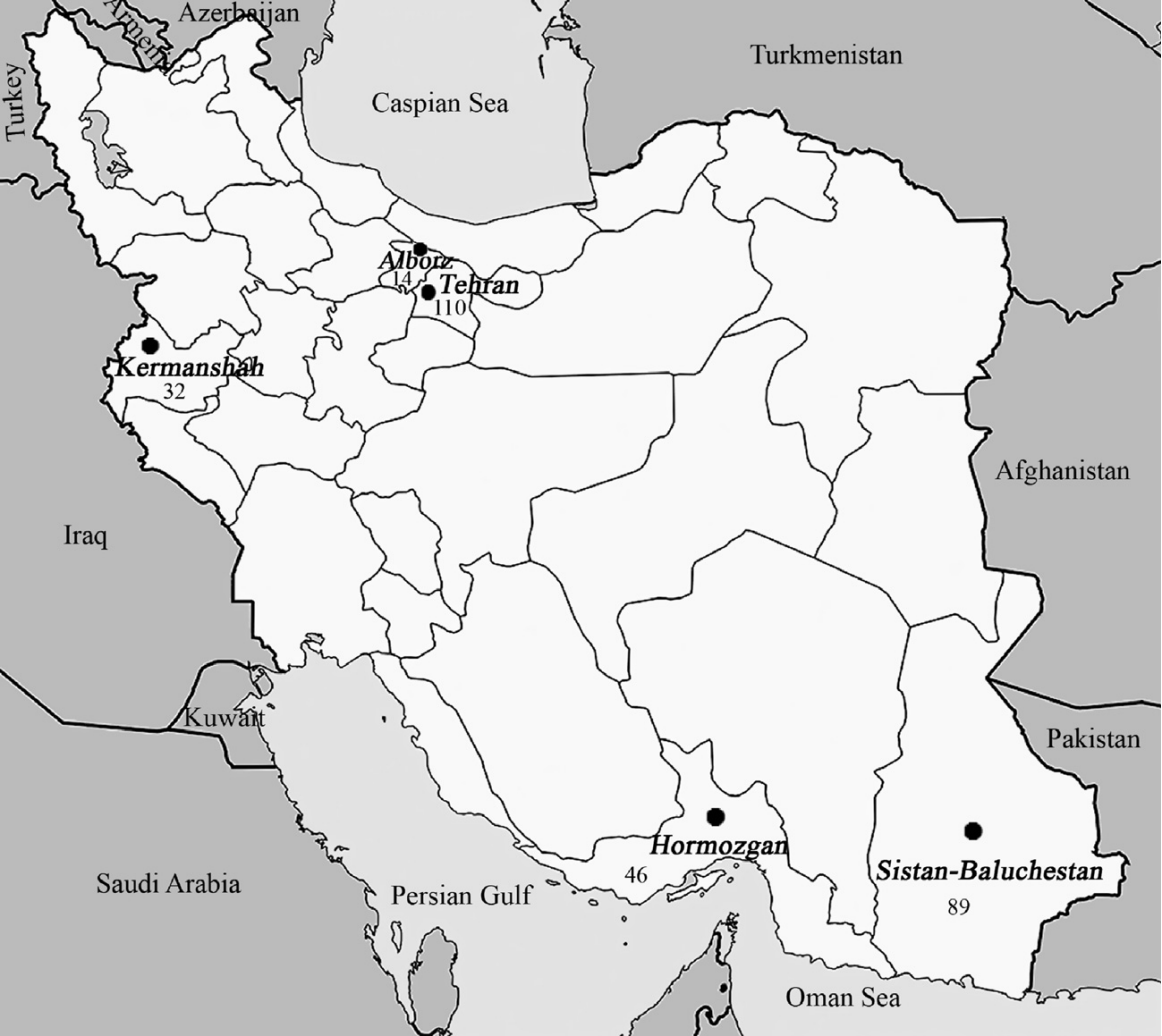
A map of Iran representing five different provinces of the country where *Mycobacterium tuberculosis* clinical isolates were obtained (The numbers indicate the number of isolates per province).

This study was conducted at the Department of Microbiology, Tehran University of Medical Sciences, Tehran, Iran. A total of 291 *M. tuberculosis* strains cultured from the same number of TB patients were included in this study. The isolates were obtained on a survey between November 2010 to July 2012 from residents of the five different provinces of the country including Tehran (*n* = 110), Alborz (*n* = 14) Sistan-Baluchestan, (*n* = 89), Hormozgan (*n* = 46), and Kermanshah (*n* = 32). Location of these provinces is shown on a map of Iran (Fig. 1). In total, 270 isolates were from pulmonary sites (Sputum [*n* = 165] and Bronchoalveolar lavage [*n* = 105]) while 21 isolates were from extrapulmonary sites (Pleural fluid [*n* = 5], wound [*n* = 8], urine [*n* = 5], and cerebrospinal fluid [*n* = 3]). Sample processing was performed based on specimen type. Nonsterile samples were digested and decontaminated with N-acetyl-L-cycteine sodium hydroxide (NALC-NaOH) method (Kubica et al. 1963; Kent and Kuniba 1985). In brief, 10–15 mL of specimen was mixed with equal volume of NALC–NaOH solution and homogenized with a vortex mixer for 15–20 sec. After incubation for 15 min, the mixture was neutralized with phosphate buffer, pH 6.8 and concentrated by centrifugation at 3000*g* for 15 min. The sediment was resuspended in 0.5 mL of sterile phosphate buffer, pH 6.8 and cultured on Lowenstein-Jensen (LJ) slant. Sterile specimens were processed and cultured without prior decontamination. LJ slants were incubated for up to 8 weeks. *M. tuberculosis* isolates were identified using standard biochemical tests, including catalase activity, production of niacin, nitrate reduction, pigment production, and growth rate (Kubica 1973).

### Drug susceptibility testing

Drug susceptibility testing against first-line drugs was performed according to proportion method on LJ media with the following drug concentrations: isoniazid (INH), 0.2 μg/mL; rifampicin (RF), 40 μg/mL; streptomycin (SM) 4 μg/mL, and ethambutol (ETB) 2.0 μg/mL (Canetti et al. 1963, 1969). Briefly, *M. tuberculosis* colonies were placed in thick-walled screw-capped glass tube containing 10 sterile glass beads (3 mm in diameter). Tubes were shaken for 20–30 sec. Distilled water was added slowly until the opacity of the bacterial suspension matched with that of 1 mg/mL of Mycobacterium bovis Bacillus Calmette-Guerin (BCG). Ten-fold serial dilutions were prepared down to a dilution of 10^−5^ and 0.1 mL of dilutions 10^−3^ and 10^−5^ was inoculated on to the control (drug-free) and drug-containing media. The extent of growth in the absence or presence of drug was compared and expressed as a percentage. Strains were considered as sensitive when the growth rate was <1% in comparison to control. MDR was defined as resistance to at least INH and RF (WHO 2010). This project has been approved by Tehran University of Medical Sciences Ethical Committee (Project No. 15356).

### DNA extraction and spoligotyping

Deoxyribonucleic acid was extracted from mycobacterial colonies growing on LJ medium using the method of Wilson (1987). Spoligotyping was performed as previously described by Kamerbeek et al. (1997) using membrane provided from National Tuberculosis Reference Laboratory, Bilthoven, the Netherlands. In brief, DR region of mycobacterial genome was amplified using PCR with primers DRa 5′ - GGT TTT GGG TCT GAC GAC -3′ (biotinylated at 5′ end) and DRb 5′-CCG AGA GGG GAC GGA AAC-3′ (Metabion, Martinsried, Germany). The PCR products were subsequently hybridized to a set of 43 oligonucleotides covalently bound to a membrane. The hybridized PCR products were incubated with streptavidin-peroxidase conjugate (Roche, Mannheim, Germany) and then detected by chemiluminescence system (Amersham ECL detection kit; GE Healthcare Limited, Little Chalfont, Buckinghamshire, U.K). The X-ray film was developed using standard photochemical procedures after 1–30 min exposure. Two membranes were purchased for genotyping of 291 samples. All samples were genotyped in duplicate by using two different membranes. For repeated use of membranes each membrane was regenerated eight to nine times by two times incubation in 1% sodiumdodecyl sulphate (SDS) at 80°C for 30 min, and subsequent washing in 20 mmol/L ethylenediaminetetraacetic acid for 15 min at room temperature (Kamerbeek et al. 1997). DNA extracts of *M. tuberculosis* H37Rv and *M. bovis* BCG were used as positive controls.

### Spoligotyping data analysis

We compared our spoligotyping results in binary format with the SITVIT2 database (Pasteur Institute of Guadeloupe) an updated version of the international spoligotyping database, SpolDB4.0 (Brudey et al. 2006) (available online at http://www.pasteur-guadeloupe.fr:8081/SITVITDemo). A spoligotype-based dendrogram was generated by the unweighted pair group method with arithmetic averages (UPGMA) calculation. A cluster was defined as two or more isolates from different patients with identical spoligotype patterns. We defined unique (nonclustered) spoligotypes as those which did not cluster with any other sample in our study. Orphan was defined as a unique spoligotype pattern not described in the SITVIT2 database. Statistical analysis was performed using chi-square test to determine statistical associations between strain types and specific parameters and values of *P* < 0.05 were considered statistically significant.

## Results

### Spoligotyping analysis

**Figure 2:**
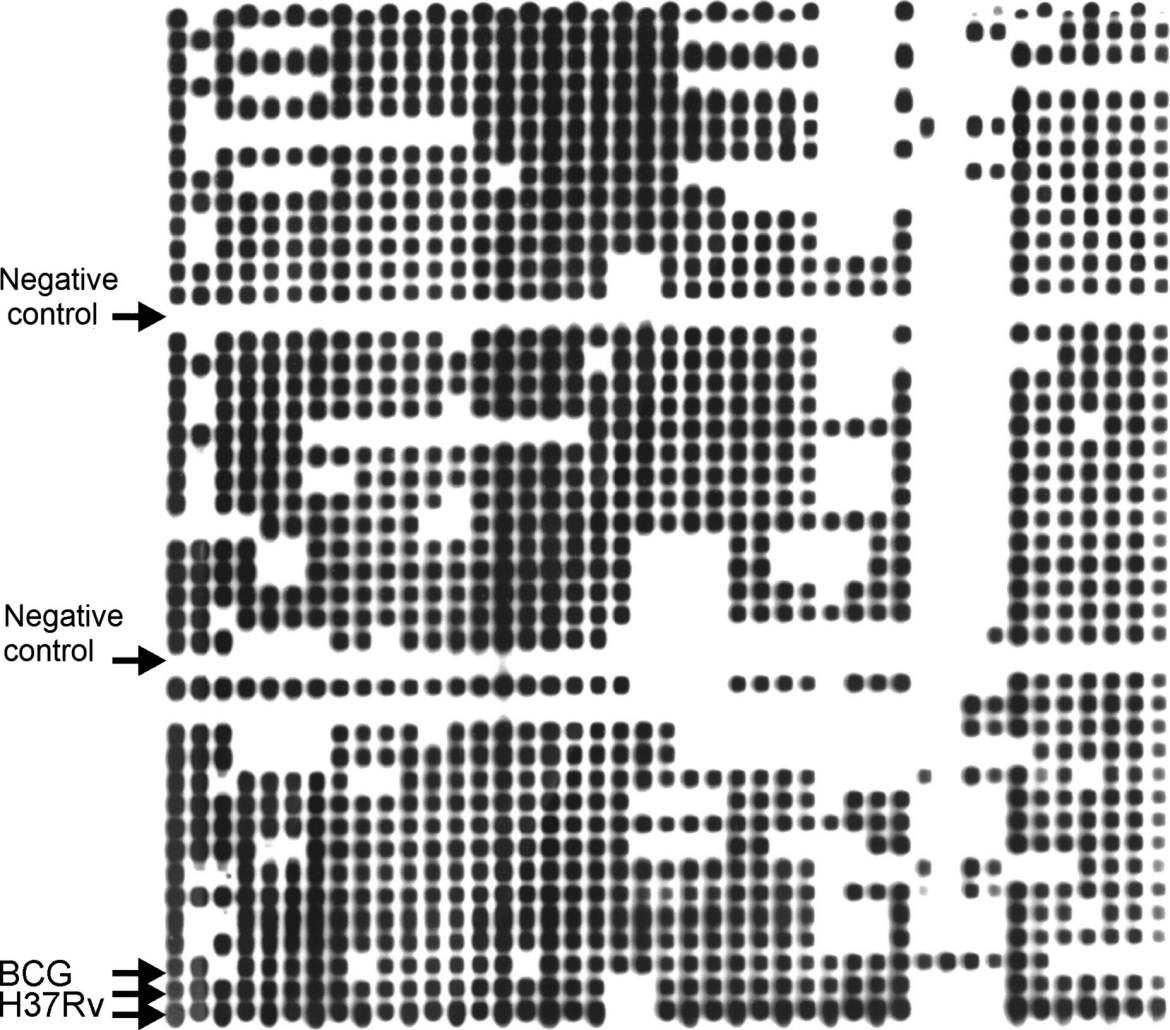
Spoligotyping autoradiograph representing hybridization patterns of amplified DNA samples from 39 *Mycobacterium tuberculosis* clinical isolates, 2 *M. tuberculosis* H37Rv and 1 *Mycobacterium bovis* BCG.

**Figure 3:**
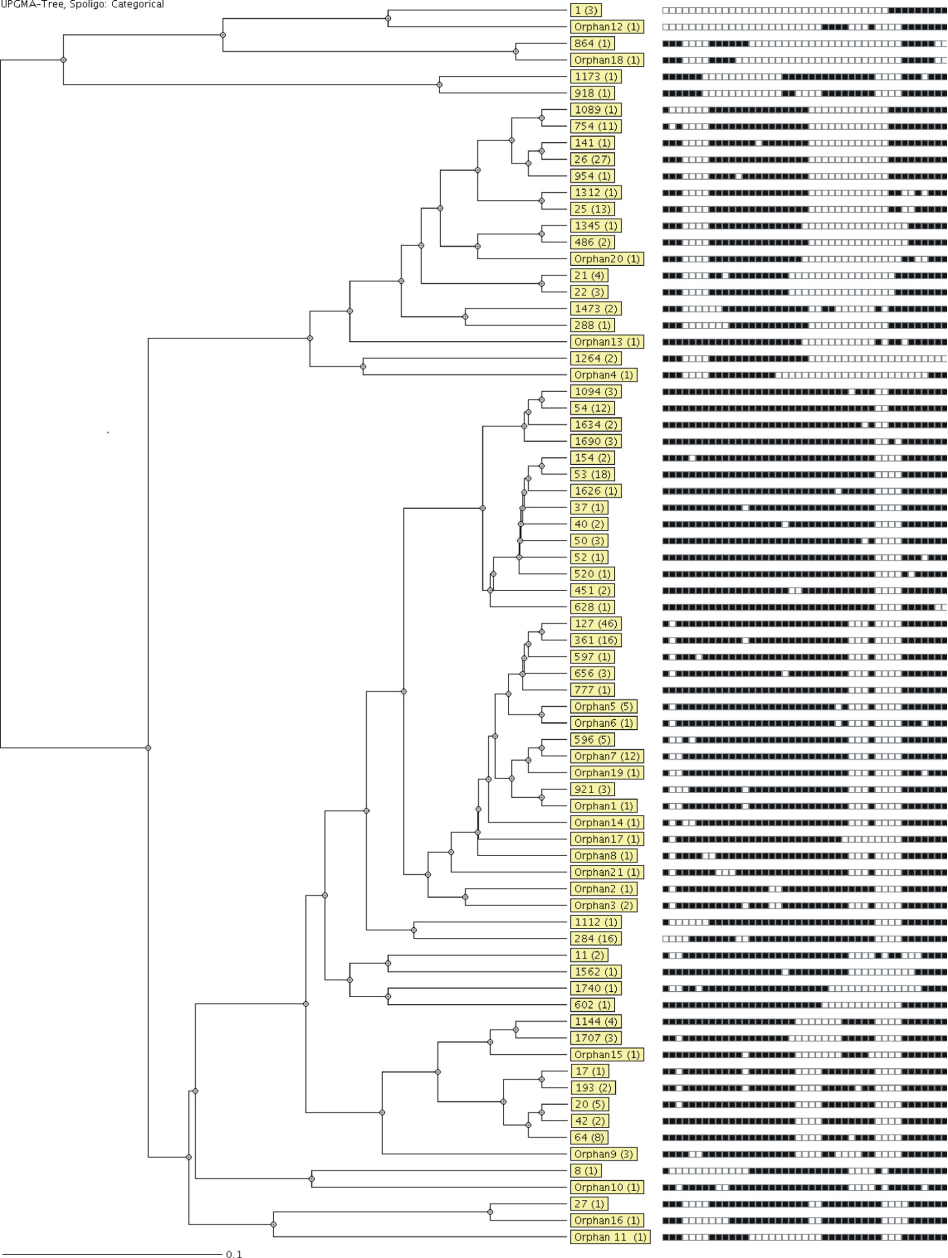
Spoligotype-based dendrogram of Iranian isolates (*n* = 291). Boxes with yellow background show spoligotypes SIT number and number of isolates enclosed in parentheses.

Spoligotyping of 291 isolates produced 75 distinct spoligotype patterns. Overall, 251 isolates (86.2%) were classified into 35 clusters containing 2–46 isolates while the remaining 40 isolates (13.7%) did not form clusters (unique patterns) (Table 1). Spoligotyping grouped 278 (95.5%) isolates in 10 families including Ural (former H4 sub-lineage, *n* = 100, 34.3%), Central Asian strain (CAS) (CAS1 and CAS2, *n* = 70, 24%), T (T1, T2, T3 and T4, *n* = 53, 18.2%), Manu2 (*n* = 22, 7.5%), Latin American-Mediterranean (LAM) (LAM1, LAM2, LAM6, LAM9, *n* = 18, 6.1%), East African Indian (EAI) (*n* = 4, 1.3%), Beijing (*n* = 3, 1%), U (*n* = 3, 1%), Haarlem (H3, *n* = 3, 1%), and H37Rv (*n* = 2, 0.6%). Twenty-one spoligotype patterns (*n* = 39 isolates) were defined as orphan, among them orphan 1, 3, 5, 6, 7, 8, 14, 19, and 21 (*n* = 25 isolates) showed high similarity to Ural genotype and were classified with Ural linage. Similarly, orphan spoligotype 10 (*n* = 1 isolate) was classified as EAI family based on high similarity to this family. The remaining 11 orphan spoligotypes (including orphan 2, 4, 9–13, 15–18 and 20, *n* = 13 isolate) were not found in the database SITVIT2 and did not show similarity to any other known lineages and were considered as possibly novel genotypes. Table 2 depicts the spoligotypes of 10 most prominent spoligotypes with their SIT number in SITVIT2 database. Table 3 shows the spoligotype description of 39 orphan isolates obtained in this study. The most frequent spoligotype in our population was Ural/SIT127 (*n* = 46, 15.8%) followed by CAS1/SIT26 (*n* = 27, 9.2%), T1/SIT53 (*n* = 18, 6.1%), T1/SIT284 (*n* = 16, 5.4%), CAS1/SIT25 (*n* = 13, 4.4%), and Manu2/SIT54 (*n* = 12, 4.1%). An autoradiograph of spoligotyping representing hybridization pattern of amplified DR locus from *M. tuberculosis* isolates is depicted in Figure 2. The UPGMA tree of *M. tuberculosis* genotypes is illustrated in Figure 3. A comparison of genotype distribution among five provinces indicated that prevalence of Ural family (*n* = 58/124, 49.7%) was higher in Tehran and Alborze, while CAS superfamily was more frequent (*n* = 40/89, 45%) in Sistan-Baluchestan. T (*n* = 16/32, 50%) and LAM (12/46, 26%) clades were prominent in Kermanshah and Hormozgan, respectively. Overall, three Beijing strains (SIT1) were identified consisting of about 1% of the isolates studied. Two of these isolates were from Tehran province and one was from Hormozgan province.

**Table 1:** Spoligoptyes distribution in clustered and nonclustered groups.

Spoligotypes	Patterns	No. of isolates (%)
Previously identified spoligotypes	54	252 (86.6)
Clusters	31	229 (78.6)
Unique	23	23 (7.9)
Orphan spoligotypes	21	39 (13.4)
Clusters	4	22 (7.5)
Unique	17	17 (5.8)
Total clusters	35	251 (86.2)
Total unique	40	40 (13.7)
Total	75	291

**Table 2:** Spoligotypes of 10 most prevalent genotypes of Iran.

SIT^1^	Family^2^	No. (%)^3^	Spoligotype pattern^4^
127	Ural	46 (15.8)	▪□▪▪▪▪▪▪▪▪▪▪▪▪▪▪▪▪▪▪▪▪▪▪▪▪▪▪□□□▪□□□□▪▪▪▪▪▪▪
26	CAS1	27 (9.2)	▪▪▪□□□□▪▪▪▪▪▪▪▪▪▪▪▪▪▪▪□□□□□□□□□□□□▪▪▪▪▪▪▪▪▪
53	T1	18 (6.1)	▪▪▪▪▪▪▪▪▪▪▪▪▪▪▪▪▪▪▪▪▪▪▪▪▪▪▪▪▪▪▪▪□□□□▪▪▪▪▪▪▪
284	T1	16 (5.4)	□□□□▪▪▪▪▪▪▪□□▪▪▪▪▪▪▪▪▪▪▪▪▪▪▪▪▪▪▪□□□□▪▪▪▪▪▪▪
25	CAS1	13 (4.4)	▪▪▪□□□□▪▪▪▪▪▪▪▪▪▪▪▪▪▪▪□□□□□□□□□□□□▪▪□□▪▪▪▪▪
54	Manu2	12 (4.1)	▪▪▪▪▪▪▪▪▪▪▪▪▪▪▪▪▪▪▪▪▪▪▪▪▪▪▪▪▪▪▪▪□□▪▪▪▪▪▪▪▪▪
New	Ural (Orphan7)	12 (4.1)	▪□□▪▪▪▪▪▪▪▪▪▪▪▪▪▪▪▪▪▪▪▪▪▪▪▪▪□□□▪□□□□▪▪▪▪▪▪▪
754	CAS1	11 (3.7)	▪□▪□□□□▪▪▪▪▪▪▪▪▪▪▪▪▪▪▪□□□□□□□□□□□□▪▪▪▪▪▪▪▪▪
64	LAM6	8 (2.7)	▪▪▪▪▪▪▪▪▪▪▪▪▪▪▪▪▪▪▪▪□□□□▪▪▪▪□▪▪▪□□□□▪▪▪▪▪▪▪
20	LAM1	5 (1.7)	▪▪□▪▪▪▪▪▪▪▪▪▪▪▪▪▪▪▪▪□□□□▪▪▪▪▪▪▪▪□□□□▪▪▪▪▪▪▪

1 SIT from SITVIT2.

2 Representing spoligotype families annotated in SITVIT2.

3 Number of strains.

4 (▪) Presence of spacer; (□) absence of spacer.

**Table 3:** Spoligotype patterns of orphan isolates of *Mycobacterium tuberculosis* in Iran.

Name of spoligopattern	No. of isolates (%)	Spoligotype pattern
Orphan seri1^1^		
Orphan1	1 (0.3)	▪□□▪▪▪▪▪▪▪▪▪□▪▪▪▪▪▪▪▪▪▪▪▪▪▪▪□□□▪□□□□▪▪▪▪▪▪▪
Orphan3	2 (0.6)	▪□▪▪▪▪▪▪▪▪▪▪□▪▪▪□□▪▪▪▪▪▪▪▪▪▪□□□▪□□□□▪▪▪▪▪▪▪
Orphan5	5 (1.7)	▪□▪▪▪▪▪▪▪▪▪▪▪▪▪▪▪▪▪▪▪▪▪▪▪▪□▪□□□▪□□□□▪▪▪▪▪▪▪
Orphan6	1 (0.3)	▪□▪▪▪▪▪▪▪▪▪▪▪▪▪▪▪▪▪▪▪▪▪▪▪▪□▪□□□▪□□□□▪▪▪□▪▪▪
Orphan7	12 (4.1)	▪□□▪▪▪▪▪▪▪▪▪▪▪▪▪▪▪▪▪▪▪▪▪▪▪▪▪□□□▪□□□□▪▪▪▪▪▪▪
Orphan8	1 (0.3)	▪□▪▪▪▪□□▪▪▪▪▪▪▪▪▪▪▪▪▪▪▪▪▪▪▪▪□□□▪□□□□▪▪▪▪▪▪▪
Orphan14	1 (0.3)	▪□▪□□▪▪▪▪▪▪▪▪▪▪▪▪▪▪▪▪▪▪▪▪▪▪▪□□□▪□□□□▪▪▪▪▪▪▪
Orphan19	1 (0.3)	▪□□▪▪▪▪▪▪▪▪▪▪▪▪▪▪▪▪▪▪▪▪▪▪▪▪▪□□□▪□□□□▪▪▪□▪▪▪
Orphan21	1 (0.3)	▪□▪▪▪▪▪▪□□□▪▪▪▪▪▪▪▪▪▪▪▪▪▪▪▪▪□□□▪□□□□▪▪▪▪▪▪▪
Orphan seri2^2^		
Orphan10	1 (0.3)	▪▪□▪▪▪▪▪□□▪▪▪▪▪▪▪▪▪▪▪▪▪▪▪▪▪▪□□□□▪□▪▪▪▪▪□▪▪▪
Orphan seri3^3^		
Orphan2	1 (0.3)	▪□▪▪▪▪▪▪▪▪▪▪▪▪▪▪□□▪▪▪▪▪▪▪▪▪▪▪▪▪▪□□□□▪▪▪▪▪▪▪
Orphan4	1 (0.3)	▪▪▪□□□□▪▪▪▪▪▪▪▪▪▪□□□□□□□□□□□□□□□□□□□□□□□▪▪▪
Orphan9	3 (1)	▪▪▪▪□□▪▪▪▪▪▪▪▪▪▪▪▪▪▪□□□□▪▪□□□□▪▪□□□□▪▪▪▪▪▪▪
Orphan 11	1 (0.3)	▪▪▪□□□□▪▪▪▪▪▪□□□□□□□□□□□▪▪▪▪▪▪▪▪▪□□□▪▪▪▪▪▪▪
Orphan12	1 (0.3)	□□□□□□□□□□□□□□□□□□□□□□□□▪▪▪▪□□□▪□□□□▪▪▪▪▪▪▪
Orphan13	1 (0.3)	▪▪▪▪▪▪▪▪▪▪▪▪▪▪▪▪▪▪▪▪▪□□□□□□□□□□□▪□▪▪□▪▪▪▪▪▪
Orphan15	1 (0.3)	▪▪▪▪▪▪▪▪▪▪▪▪□▪▪▪▪▪▪▪□□□□□□□▪▪▪▪□□□□□▪▪▪▪▪▪▪
Orphan16	1 (0.3)	▪▪▪□□□□□□□▪▪▪▪▪▪▪▪▪▪▪▪□□▪▪▪▪▪▪▪▪▪□□□□▪▪▪▪▪▪
Orphan17	1 (0.3)	▪□▪▪▪▪▪▪▪▪▪▪▪▪▪▪▪▪▪▪▪▪▪▪▪▪▪□□□□□□□□□▪▪▪▪▪▪▪
Orphan18	1 (0.3)	▪▪▪□□□□▪▪▪▪□□□□□□□□□□□□□□□□□□□□□□□□□▪▪▪▪▪□□
Orphan20	1 (0.3)	▪▪▪□□□□▪▪▪▪▪▪▪▪▪▪▪▪▪▪□□□□□□□□□□□□□□□▪▪□□▪▪▪

1 Orphan spoligotypes related to Ural lineage.

2 Orphan spoligotype related to EAI lineage.

3 Orphan spoligotypes not identified as genotype of any phylogenetic lineage.

### Patient characteristics

A total of 291 *M. tuberculosis* strains were isolated from 135 women aged between 9 and 88 and 156 men aged between 2 and 89. The average age of studied group was 56 years. Patients in the group aged >50 years had the highest number (52%, *n* = 152) of isolates, followed by those aged between 30 and 50 with 30.5% (*n* = 89) of the isolates.

### Drug resistance patterns

Of the 291 strains in this study 84.8% (*n* = 247) were sensitive to all tested agents. Among the 44 drug-resistant isolates, 15 (34%) were MDR and remaining 29 isolates showed mono drug resistance for INH (*n* = 4), RF (*n* = 2), SM (*n* = 21), and ETB (*n* = 2). 7.5% (*n* = 3/40) of nonclustered isolates were drug-resistant compared with 16.3% (*n* = 41/251) of clustered isolates. This difference was not statistically significant (*P-*value = 0.061 [Pearson's chi-square test]).

### Association between genotype and drug resistance phenotype

In an investigation between *M. tuberculosis* genotypes and drug resistance we found that Beijing genotype was highly associated with MDR. Actually, all three Beijing strains showed MDR phenotype. Only 5% of Ural family isolates were MDR in this study. This figure is not significantly different from that of other circulating genotypes in our setting (*P* = 0.99, chi-square test) (Table 4).

**Table 4:** Association between bacterial genotype and drug resistance.

	No. of strains (%)					
	
Characteristic	Ural	CAS	Beijing	Orphan	Others^1^	All spoligotypes
INH resistance	3 (3)	0	0	0	1 (0.9)	4 (1.3)
RF resistance	1 (1)	0	0	0	1 (0.9)	2 (0.6)
SM/ETB resistance	12 (12)	4 (5.7)	0	0	7 (6.6)	23 (8)
MDR	5 (5)	1 (1.4)	3 (100)	0	6 (5.7)	15 (5)
Sensitivity to all drugs	79 (79)	65 (92.8)	0	13 (100)	90 (85.7)	247 (84.8)
Total	100	70	3	13	105	291

1 Includes T, Manu2, LAM, EAI, U, Haarlem, and H37Rv families.

## Discussion

In this study, we provided information about the genotypic biodiversity and drug resistance of *M. tuberculosis* strains collected from five provinces of Iran. About 86.2% of our isolates were clustered by spoligotyping. Previous spoligotyping studies performed in Iran (Farnia et al. 2006; Ramazanzadeh et al. 2009) showed that Haarlem was the most frequent lineage. East African Indian was the most predominant family in another study reported from Iran (Velayati et al. 2006). However, the majority of *M. tuberculosis* strains presented in this study was characterized with Ural family. Ural (former H4 sub-lineage) lineage has been recently excluded from the Haarlem family based on not harboring *mgtC545*(CGC → CAC) mutation (Abadia et al. 2010). The CAS family was ranked as the second most prevalent spoligotype. It is overrepresented by prototype SIT26 and was causative of TB in 24% of the cases. Principally, 57% (*n* = 40/70) of this family were from Sistan-Baluchestan which is the largest province of Iran with the highest rate of TB in the country. This province is located in the southeast of the country and shares geographic borders with Afghanistan and Pakistan where the CAS family is dominant. In its recent history Iran has been ranked as world's largest refugee haven, mainly for Afghans and Iraqis. Iran also hosts some refugees of various nationalities, the majority of these being Pakistanis, Afghans, Tajiks, Bosnians, and Azeris. Also, at the end of 2005, the UN Refugee Agency estimated that Iran was host to the third largest refugee population in the world, with a total of 716,000 refugees (http://www.migrationinformation.org). Because of the high prevalence of CAS family (especially SIT26) in Pakistan (Hasan et al. 2006; Tanveer et al. 2008) it is possible that this family strains were transported from Pakistan to Sistan-Baluchestan. However, the proof of this hypothesis is difficult as more population-based epidemiologic studies are required. The prevalence of Beijing strains shows geographic variation. High rates of Beijing genotype have been reported from Asian countries (China, Hong Kong, and Thailand >50%) (Glynn et al. 2002), while in Western European and Czech Republic (<6%) and Latin America (<1%) its prevalence is low (European Concerted Action on New Generation Genetic Markers and Techniques for the Epidemiology and Control of Tuberculosis 2006). Previous research from Iran showed that the percentage of Beijing genotype was 20.5% (Farnia et al. 2006). However, this genotype comprised only 1% of our studied strains. The explanation for this difference may be that the strains used in the previous study were obtained from patients with MDR-TB which is highly associated with Beijing genotype. Most *M. tuberculosis* isolates recovered in Iran were susceptible to all tested antituberculosis drugs. In this study, about 10% of the isolates showed mono drug resistance and 5% of isolates were MDR. Association between Beijing genotype and MDR-TB varies worldwide. While researches from Russia, Cuba (Glynn et al. 2002), and Pakistan (Tanveer et al. 2008) reported such an association, it has not been noted in reports from Indonesia, Western Sweden (Brudey et al. 2004), and Hong Kong (Glynn et al. 2002). Analysis of association between bacterial genotype and drug resistance showed relationship between Beijing isolates and MDR-TB. However, the proportion of this genotype in this study population is too small. Therefore, a greater study is required to confirm this finding. In spite of the high prevalence, only five out of the 100 (5%) Ural type isolates were MDR in this study. This prevalence was not significantly different from that of the other circulating genotypes in our population. The third most predominant family in this study was ill-defined T family (in which spacers 33–36 are absent), which is also reported from western Sweden (Brudey et al. 2004), Brazil (Mendes et al. 2011) and Pakistan (Tanveer et al. 2008). The strains of this family comprised about 18% of all isolates and 50% of isolates from Kermanshah Province. This province is a part of region called Iranian Kurdistan, which is an unofficial name for the parts of Iran inhabited by Kurdish people and has borders with Iraq and Turkey. In several spoligotyping studies carried out in Turkey (Gencer and Shinnick 2005; Kisa et al. 2007; Aktas et al. 2008) ill-defined T family (especially SIT53) was reported to be the most frequent genotype. This family was demonstrated as the predominant family in a study performed in Iraq (Merza et al. 2011). This correlation suggests a possible role for migration, trade and tourism in distribution of T family among these three countries (“Turkey, Iran ready to bolster tourism” Turkish daily news. 19 June 2006; http://www.thenational.ae/business/travel-tourism/iraqi-citys-religious-tourism-set-to-grow).

In conclusion, this study gives preliminary information about major *M. tuberculosis* strains circulating in Iran and their drug resistance pattern. The main Iranian families found in this study were Ural, CAS, and T. All Beijing strains were MDR, whereas the most frequent lineage, Ural did not show significant association with antituberculosis drug resistance. We suggested that CAS and T families may be transmitted from bordering provinces of the Iran to other regions of the country. Genotyping with other powerful methods and studying other provinces in Iran will be the next step of this ongoing work to provide better understanding of the epidemiology of TB within Iran.
